# Combining Mobilizing Agents with Busulfan to Reduce Chemotherapy-Based Conditioning for Hematopoietic Stem Cell Transplantation

**DOI:** 10.3390/cells10051077

**Published:** 2021-04-30

**Authors:** Laura Garcia-Perez, Lieke van Roon, Marco W. Schilham, Arjan C. Lankester, Karin Pike-Overzet, Frank J. T. Staal

**Affiliations:** 1 Department of Immunology, Leiden University Medical Center, 2333 ZA Leiden, The Netherlands; l.garcia@lumc.nl (L.G.-P.); l.van_roon@lumc.nl (L.v.R.); k.pike-overzet@lumc.nl (K.P.-O.); 2 Department of Pediatrics, Willem-Alexander Children’s Hospital, Leiden University Medical Center, 2333 ZA Leiden, The Netherlands; m.w.schilham@lumc.nl (M.W.S.); a.lankester@lumc.nl (A.C.L.)

**Keywords:** conditioning, busulfan, G-CSF, plerixafor, HSC transplantation, immune reconstitution

## Abstract

In the context of hematopoietic stem cell (HSC) transplantation, conditioning with myelo- and immune-ablative agents is used to eradicate the patient’s diseased cells, generate space in the marrow and suppress immune reactions prior to the infusion of donor HSCs. While conditioning is required for effective and long-lasting HSC engraftment, currently used regimens are also associated with short and long-term side effects on extramedullary tissues and even mortality. Particularly in patients with severe combined immunodeficiency (SCID), who are generally less than 1-year old at the time of transplantation and often suffer from existing comorbidities. There is a pressing need for development of alternative, less toxic conditioning regimens. Hence, we here aimed to improve efficacy of currently used myeloablative protocols by combining busulfan with stem-cell niche-directed therapeutic agents (G-CSF or plerixafor) that are approved for clinical use in stem cell mobilization. T, B and myeloid cell recovery was analyzed in humanized NSG mice after different conditioning regimens. Increasing levels of human leukocyte chimerism were observed in a busulfan dose-dependent manner, showing comparable immune recovery as with total body irradiation in CD34-transplanted NSG mice. Notably, a better T cell reconstitution compared to TBI was observed after busulfan conditioning not only in NSG mice but also in SCID mouse models. Direct effects of reducing the stem cell compartment in the bone marrow were observed after G-CSF and plerixafor administration, as well as in combination with low doses of busulfan. Unfortunately, these direct effects on the stem population in the bone marrow were not reflected in increased human chimerism or immune recovery after CD34 transplantation in NSG mice. These results indicate moderate potential of reduced conditioning regimens for clinical use relevant for all allogeneic transplants.

## 1. Introduction

Allogeneic and gene-corrected autologous hematopoietic stem cell (HSC) transplantation may result in limited engraftment of progenitors without a preceding conditioning regimen due to the occupation of bone marrow (BM) and thymic niches by host cells, which results in incomplete graft function, immune reconstitution and cure [1]. Conditioning agents can be employed to create space in the BM niches thus allowing transplanted HSCs to engraft efficiently. Although conditioning contributes to an improved HSC transplantation outcome by increasing HSC engraftment, immune chimerism and immune function and by reducing the risk of graft rejection, it may also have a negative impact on patient well-being due to short-term and long-term treatment-related morbidity and mortality [2,3]. The use of irradiation-based regimens and alkylating chemotherapy in infants has an unfavorable impact on growth and fertility, and is associated with an increased risk of secondary malignancies [4,5,6]. Therefore, particularly in pediatric patients, total body irradiation regimens have been gradually replaced by chemotherapy-based conditioning [7]. Busulfan is a myeloablative alkylating agent that prevents DNA replication through DNA crosslinking and therefore triggering cell apoptosis [8]. Busulfan is used as a conditioning agent prior to HSC transplantation as it is known to be cytotoxic to host hematopoietic stem and progenitor cells (HSPCs) [9].

In the first stem cell gene therapy protocols for severe combined immunodeficiency (SCID), conditioning was omitted. The absence of conditioning prior to both allogeneic and gene-corrected autologous HSC transplantation led to limited engraftment of transplanted HSCs and thus only partial correction of the immune deficiency, especially B cell function, resulting in suboptimal clinical benefit. [3,10]. Subsequent clinical trials of gene therapy for SCID included the use of a non-myeloablative reduced-intensity conditioning (RIC) regimen consisting of low-dose busulfan-based conditioning (4 mg/kg) with approximately 25% of the total dose usually used in myeloablative protocols. The use of RIC regimens enables the engraftment of early progenitor cells and therefore allows both T- and B-cell long-term correction, while limiting potential short- and long-term toxicities [11,12,13]. However, insufficient conditioning is associated with the risk of mixed chimerism in the HSC compartment [14] and therefore reduces the chance of a favorable outcome. Current gene therapy protocols for SCID, especially ADA-SCID and X-linked SCID, rely on the use of HSC corrected cells and a reduced-intensity busulfan-based conditioning regimen, which have been shown to be successful in achieving a lasting effective engraftment with limited toxicity [11,15,16].

However, this reduced-intensity busulfan-based conditioning may be insufficient in other forms of SCID, such as the recombinase-activating gene 1 and 2 (RAG1/2) SCID where there is a more prominent occupancy of BM niches by precursor B cells blocked in development. For this patient group, insufficient HSC engraftment, resulting in poorer T- and B-cell reconstitution, has been reported in the absence of conditioning. [17,18,19]. In RAG1/2 SCID, precursor B cells completely occupy bone marrow niches and strongly compete with transplanted cells, leading to poor immune reconstitution [20,21]. Therefore, to achieve proper engraftment of transplanted cells, a myeloablative regimen is required to empty precursor niches. Conditioning benefits should also be weighed against its short- and long-term toxicity, especially in for instance Artemis deficiency with inherent radiosensitivity due to impaired DNA repair and in newborn patients [3,6]. Accordingly, a critical balance for successful engraftment together with the risk of dose-limiting toxicities must be carefully considered and highlights the need to develop alternative non-/less genotoxic conditioning regimens.

Thus, although current conditioning agents are often successfully employed, there is a pressing need for alternative, less toxic conditioning regimens to create space in the BM niches for a durable engraftment of stem cells/HSC without adverse effects on extramedullary tissues. Development of effective, nontoxic, non-alkylating-based conditioning regimens is essential to ensure a successful transplantation and good quality of life in patients with SCID or related inborn errors. In SCID, where patients are predominantly less than 1-year old at the time of treatment and where comorbidities including viral infections are frequently present, reducing conditioning-related toxicity and improving the rate of immune recovery are of great importance.

Hence, we here explored alternative approaches including the added value of clinically approved mobilizing agents such as G-CSF (granulocyte-colony stimulating factor) or plerixafor. G-CSF together with plerixafor is used to mobilize HSCs from the BM niche to the bloodstream for HSC collection in autologous transplants. G-CSF mobilizes by impairing HSC niche function in the BM by suppressing niche-supportive cells and cytokines whereas plerixafor (also known as AMD3100) directly targets HSC without altering HSC niche function by directly antagonizing the CXCR4-mediated sensing that retains HSCs within the BM [22]. Therefore, we studied whether combining chemotherapy regimens similar to those used in clinical setting with stem cell niche-directed therapeutic agents (HSC mobilizing agents) would result in engraftment of transplanted progenitor cells with equivalent efficacy at lower chemotherapy exposure in comparison with current standard chemotherapy-based conditioning. With this aim we first assessed the efficacy and tolerability of busulfan conditioning in mice. Secondly, we examined the direct effect of the chemotherapy and the HSC mobilizing agents in the BM and the HSC niches. Finally, we analyzed whether alternative low-toxicity conditioning regimens allowed successful and equivalent immune reconstitution in NSG mice compared to a high standard chemotherapy dose.

## 2. Material and Methods

### 2.1. Human CD34+ Cell Enrichment

Human cord blood was obtained according to the medical ethical committee and IRB guidelines at Leiden University Medical Center. Cord blood mononuclear cells were separated by Ficoll (Pharmacy Leiden Academic Hospital, Leiden, The Netherlands) gradient centrifugation, frozen in fetal bovine serum (Hyclone)/10% DMSO (Sigma-Aldrich, St. Louis, MO, USA) and stored in liquid nitrogen. After thawing, human CD34+ cells were isolated using a CD34 MicroBead UltraPure Kit (Miltenyi Biotec, Bergisch Gladbach, Germany). In short, cells were incubated with FcR blocking reagent and CD34 Microbeads Ultrapure following the manufacturer’s protocol for 30 min at 4 °C. Subsequently CD34+ cells were positively selected using the appropriate ferromagnetic columns and the MACS separator (Miltenyi Biotec, Bergisch Gladbach, Germany). Hematopoietic progenitor stem cell (HSPC) count and purity after isolation were evaluated using a customized Flexicyte program on a NucleoCounter3000 (Chemometec, Allerod, Denmark). Directly isolated CD34+ cells were stimulated for 2 days in StemSpan serum-free expansion medium (StemSpan-SFEM; STEMCELL Technologies, Vancouver, BC, Canada) supplemented with 10 ng/mL human stem cell factor (huSCF; Miltenyi Biotec, Bergisch Gladbach, Germany), 20 ng/mL human thrombopoietin (huTPO; R&D Systems, Minneapolis, MN, USA), 20 ng/mL recombinant mouse insulin-like growth factor 2 (IGF2; R&D Systems, Minneapolis, MN, USA) and 10 ng/mL recombinant human fibroblast growth factor-acidic (hIFG1; PeproTech, London, UK).

### 2.2. Murine HSPC Isolation

Lineage negative depletion was performed using the Lineage Cell Depletion kit from Miltenyi Biotec, to isolate hematopoietic stem cells from frozen murine bone marrow. In short, cells were magnetically labeled with the Biotin-Antibody Cocktail and incubated for 10 min at 4 °C and subsequently incubated for 15 min at 4 °C with Anti-Biotin Microbeads. Lineage negative cells were subsequently depleted using the appropriate magnetic columns and the MACS separator (Miltenyi Biotec, Bergisch Gladbach, Germany). Directly enriched HSPCs were cultured in StemSpan (SFEM) medium supplemented with Pen/Strep (Gibco), 50 ng/mL recombinant mouse (rm) Flt3L, 100 ng/mL rmSCF and 10 ng/mL rmTPO (all from R&D Systems, Minneapolis, MN, USA) at 37 °C with 5% CO_2_. Depletion efficiency and purity of lineage negative population were analyzed by flow cytometry with BD FACS Canto II 3L (BD Biosciences, San Jose, CA, USA).

### 2.3. Mice

BALB/c Rag2/Il2rg double-knockout mice were a kind gift from Dr. E.J. Rombouts from the Department of Hematology at Erasmus MC (University Medical Center Rotterdam, Rotterdam, The Netherlands). Wild-type C57BL/6, BALB/c and NOD.Cg-Prkdc^scid^ Il2rg^tm1Wjl^/SzJ (NSG) mice were purchased from Charles River (Netherlands and France). Mice were bred and maintained in the animal facility of Leiden University Medical Center (LUMC, Leiden, The Netherlands). All animal experiments were approved by the Dutch Central Commission for Animal experimentation (AVD 1160020174064, Centrale Commissie Dierproeven, Leiden, CCD).

### 2.4. Pre-Conditioning of Mice

Rag2^−/−^ mice were conditioned with a total body single dose of irradiation 24 h prior to transplantation using orthovoltage X-rays (8.08 Gy) or with two consecutive doses of 25 mg/kg busulfan (1 mg/mL; Sigma-Aldrich) (48 h and 24 h prior to transplantation). NSG mice were conditioned with injected busulfan intraperitoneally, in a single dose (5 mg/kg, 12.5 mg/kg and 25 mg/kg) 24 h prior to cell transplantation or with 2 consecutive doses of 25 mg/kg busulfan (48 h and 24 h prior to transplantation) for the highest dose (50 mg/kg).

HSPC mobilization was performed with G-CSF (Neulasta^®^, Amgen, Thousand Oaks, CAUSA) up to a total dose of 125 µg/kg. Mice were injected subcutaneously on 2 consecutive days, 24 h apart with the last injection 24 h before the transplantation or analysis. Plerixafor (AMD3100, Sigma-Aldrich, St. Louis, MO, USA) was also used as a HSC mobilization agent. A single dose of 10 mg/kg was injected subcutaneously 1 h prior to transplantation or analysis. Preconditioning of NSG mice with the different regimens described in the paper (busulfan, G-CSF, plerixafor and combinations) were weight-adjusted per mouse.

### 2.5. HSPC Transplantation

Cells were harvested and resuspended in Iscove’s Modified Dulbecco’s Medium (IMDM) without phenol red (Gibco ThermoFisher, Waltham, MA, USA) for intravenous injection into the tail veins of preconditioned mice. Human CD34+ enriched cells (100,000 cells per mouse) were transplanted into 5−6-week-old female NSG mice, while murine HSPCs (mixed with supportive Rag2^−/−^ spleen cells (3 × 10^6^ cells/mouse) and transplanted into preconditioned Rag2^−/−^ recipient mice (8–12-week-old mice).

Mice used for transplantation were kept in a specified pathogen-free section. For the first four weeks after transplantation mice were fed with additional DietGel^®^ recovery food (clear H_2_O, Portland, ME, USA) and antibiotic water containing 0.07 mg/mL Polymixin B (Bupha Uitgeest, The Netherlands), 0.0875 mg/mL Ciprofloxacin (Bayer B.V., Mijdrecht, the Netherlands) and 0.1 mg/mL Amfotericine B (Bristol-Myers Squibb, Woerden, the Netherlands) and their welfare was monitored daily. Peripheral blood (PB) from transplanted mice was drawn by tail vein incision and analyzed every 4 weeks until the end of the experiment (20 to 24 weeks after transplantation). Peripheral blood, thymus, spleen and bone marrow were obtained from CO_2_ euthanized mice.

### 2.6. Flow Cytometry Analysis

Single cell suspensions from thymus and spleen were prepared by squeezing the organs through a 70 μM cell strainer (BD Biosciences, San Jose, CA, USA). Bone marrow single cell suspension was obtained from flushed or crushed bones (femurs and tibias) and cells were also passed through a 0.7 µm cell strainer (BD Falcon). Erythrocytes from PB and spleen were lysed using NH_4_Cl (8.4 g/L)/KHCO_3_ (1 g/L) solution (LUMC Apotheek, Leide, The Netherlands). Mononuclear cells were counted and stained with the antibodies listed in Supplementary Table S1. Briefly, cells were incubated for 30 min at 4 °C in the dark with the antibody-mix solution including directly conjugated antibodies at the optimal working solution in FACS buffer (PBS pH 7.4, 0.1% azide, 0.2% BSA). After washing with FACS buffer, a second 30 min incubation step at 4 °C was performed with the streptavidin-conjugated antibody solution. When necessary, 7AAD (BD Biosciences) was used as viability dye. Data were acquired on a BD FACS Canto II 3L and a BD FACS LSR Fortessa X-20 4L (BD Biosciences, San Jose, CA, USA) and analyzed using FlowJO software v10.6.1 (Tree Star, Ashland, OR, USA).

### 2.7. Statistics

Statistics were calculated and graphs were generated using GraphPad Prism6 (GraphPad Software). Statistical significance was determined by a one-way or two-way ANOVA test (* *p* < 0.05, ** *p* < 0.01, *** *p* < 0.001 and **** *p* < 0.0001).

## 3. Results

### 3.1. Busulfan Conditioning as an Alternative to TBI in Mice

**Figure 1:**
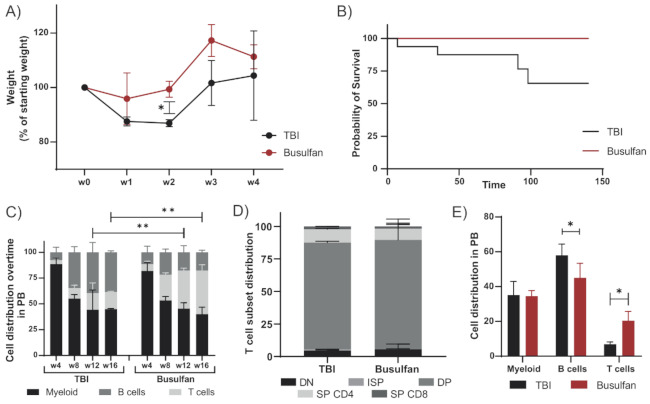
Busulfan conditioning as an alternative to TBI in immunodeficient mice: Rag2^−/−^ mice transplanted with wild-type BALB/c HSPCs were preconditioned by total body irradiation (TBI, 8.09 Gy) or busulfan (50 mg/kg). Immune reconstitution was analyzed up to 20 weeks after transplantation. (**A**) Mice were weighed weekly during the first month after transplantation. Change of weight normalized to the starting weight before conditioning is depicted in the graph for TBI (2 mice) and busulfan (3 mice) treated mice. (Unpaired t-test; * *p* < 0.05). (**B**) Survival analysis of the TBI-conditioned (16 mice from historical data) and busulfan-conditioned mice after transplantation (4 mice). (**C**) Cell distribution (myeloid, B and T cell) in peripheral blood (PB) over time of mice preconditioned with TBI (2 mice) or busulfan (4 mice). (Two-way ANOVA; ** *p* < 0.01). (**D**) Proportion of the different T-cell developmental subsets in the thymus after TBI or busulfan conditioning. (**E**) Cell distribution (myeloid, B and T cells) in PB 20 weeks after transplantation. (Two-way ANOVA; * *p* < 0.05).

The standard preconditioning method in mice for hematopoietic stem cell (HSC) transplantation is total body irradiation (TBI), varying the irradiation dose depending on the mouse strain. Rag2^−/−^ mice were transplanted with wild-type BALB/c hematopoietic and progenitor stem cells (HSPCs) after conditioning with TBI (8.09 Gy) or busulfan (50 mg/kg) as previously published for immunodeficient mice [23,24]. Improved welfare and well-being of the animals was observed for mice preconditioned with busulfan compared to TBI, with a lower loss of weight and a faster recovery after transplantation (Figure 1A). The survival rate of busulfan-conditioned mice was higher than that of TBI-treated mice (Figure 1B), which died from irradiation side effects which require strict and careful animal support and can lead to high mortality rates [24]. In addition, busulfan-conditioned mice showed increased T-cell reconstitution from week 12 after transplantation, represented by a more significant population in the peripheral blood (PB) (Figure 1C). Although T-cell development in the thymus including all development stages was comparable between busulfan- and TBI-conditioned mice (Figure 1D), the T-cell output in PB at 20 weeks after transplantation was higher for busulfan-conditioned mice (Figure 1E).

**Figure 2:**
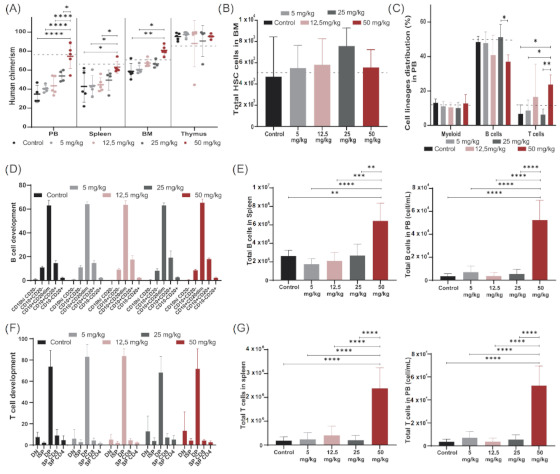
Modeling busulfan conditioning in NSG mice, determining a suitable dose. NSG mice were preconditioned with increasing doses of busulfan (control, 5 mg/kg, 12.5 mg/kg, 25 mg/kg and 50 mg/kg) and transplanted with 100,000 human CD34 cells (5 mice/group). (**A**) Human chimerism (% hCD45 cells) achieved in PB, spleen, bone marrow (BM) and thymus 20 weeks after transplantation. Human chimerism achieved by TBI represented by dashed line. (Two-way ANOVA; * *p* < 0.05, ** *p* < 0.01, *** *p* < 0.001, **** *p* < 0.0001). (**B**) Total number of human hematopoietic stem cells (HCS) in NSG BM 20 weeks after transplantation. (**C**) Cell lineage distribution (myeloid, B and T cells) in PB 20 weeks after transplantation of the different conditioned groups. (Two-way ANOVA; * *p* < 0.05, ** *p* < 0.01). (**D**) Proportion of B cell developmental stages in BM in the different busulfan-treated mice. (**E**) Total B cell counts in spleen and in PB 20 weeks after transplantation. (One-way ANOVA; ** *p* < 0.01, *** *p* < 0.001, **** *p* < 0.0001). (**F**) Proportional T-cell developmental stages in the thymus in the different busulfan-treated mice. (**G**) Total T cell counts in spleen and in PB 20 weeks after transplantation. (One-way ANOVA; **** *p* < 0.0001).

The immune outcome of busulfan-conditioned (50 mg/kg dose) NSG-transplanted mice was also comparable to that of TBI-treated NSG mice previously published [25]. Overall human engraftment, HSC engraftment in the bone marrow (BM) and immune cell distribution of mice preconditioned with a 50 mg/kg dose of busulfan (Figure 2A–C) matched TBI-conditioned reference values (horizontal black dot line).

Busulfan conditioning may lead to better conservation of tissue integrity than TBI, allowing for a higher immune output after transplantation, mainly seen in the T-cell compartments. Importantly, the welfare and well-being of the animals were improved, without compromising the overall immune recovery. Therefore, busulfan conditioning represents a favorable regimen to use in preclinical studies in mice, bringing the model a step closer towards mirroring clinical protocols.

### 3.2. Modelling Busulfan Conditioning in NSG Mice; Determining a Suitable Dose

We first focused on setting the optimal busulfan dose in NSG mice to investigate the possibility of reducing busulfan conditioning before HSC transplantation to reduce associated side effects. A dose of 50 mg/kg busulfan was used as a starting dose [23,26,27,28], and reduced gradually to 5 mg/kg. Mice were preconditioned with different doses of busulfan (control without busulfan, 5 mg/kg busulfan, 12.5 mg/kg busulfan, 25 mg/kg busulfan and 50 mg/kg busulfan as described in Material and Methods) and transplanted intravenously with 1 × 10^5^ CD34+ cells/kg isolated from cord blood (five mice/group). Human chimerism and human immune cell reconstitution were followed up to 20 weeks after transplantation (Supplementary Figure S1). Mice were sacrificed and immune organs were thoroughly analyzed for human HSC engraftment and human B- and T-cell development. Increasing levels of human chimerism were observed in PB, spleen and BM with increasing busulfan doses, with a significant increase in the group receiving the maximum dose (50 mg/kg) compared to the control group and the lower 5 mg/kg and 12.5 mg/kg doses (Figure 2A). As NSG thymi are devoid of murine cells, human engrafted cells completely repopulated the thymus in all dosing groups, showing close to 100% human chimerism in this organ. Although a comparable number of human HSCs were engrafted in BM across the groups (Figure 2B), the distribution of immune cell lineages in PB, mainly B and T cells, significantly differed for the highest dose compared to other groups, leading to a higher T-cell contribution (Figure 2C). All busulfan doses contributed to an overall normal B-cell development in BM (Figure 2D) and T-cell development in the thymus (Figure 2F) with a normal population distribution over the developmental stages. However, significantly higher B-cell (Figure 2E) and T-cell (Figure 2F,G) numbers were detected in the periphery (spleen and PB) with the highest dose, while following more moderate doses, immune output was comparable to that of control transplanted mice.

High-dose busulfan (50 mg/kg) gave reliably higher immune reconstitution and was set as the high dose for the following experiments. Consistent immune development and chimerism were detected for lower busulfan doses, and therefore we set the 12.5 mg/kg dose as our low dose of busulfan for the following experiments where we aimed to improve our low-dose busulfan immune outcome by combining with stem cell niche-directed non-chemotherapeutic agents.

### 3.3. Short-Term Effect of Busulfan and Mobilizing Agents on BM HSCs

**Figure 3:**
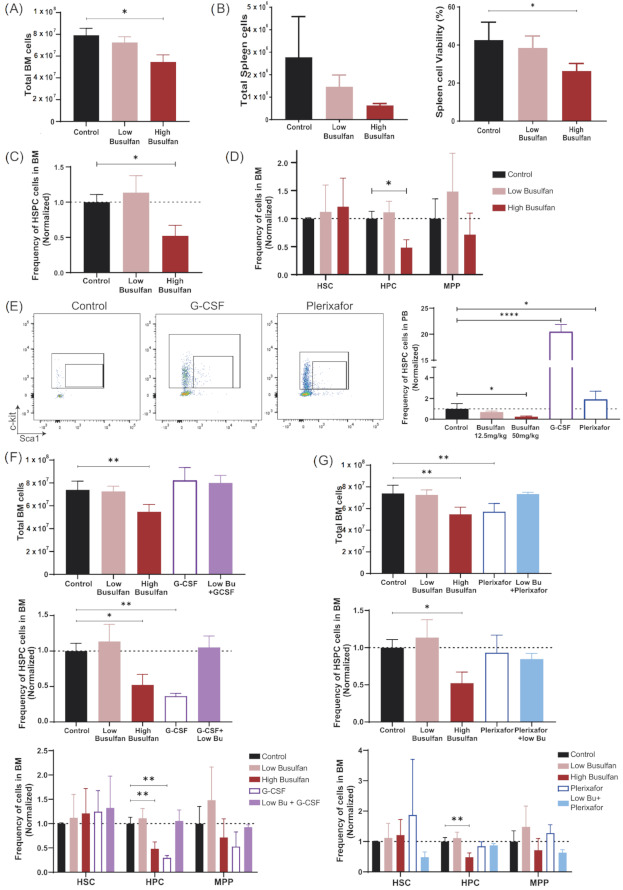
Effect of busulfan and mobilizing agents on BM HSCs. (**A**) Total BM cell numbers, 24 h after busulfan conditioning (low and high dose) compared to the control group (without busulfan). (One-way ANOVA, * *p* < 0.05). (**B**) Total spleen cell numbers and cell viability after busulfan conditioning (24 h after). (One-way ANOVA; * *p* < 0.05). (**C**) Frequency of HSPCs (LSK; Lin-Sca1 + cKit +) cells in BM 24 h after busulfan conditioning, normalized to control mice. (One-way ANOVA; * *p* < 0.05). (**D**) Frequency of long-term HSCs (Lin-Sca1+cKit+CD150+CD48-), hematopoietic progenitor cells (HPC; Lin-Sca1+cKit+CD48+) and multipotent progenitor cells (MPP; Lin-Sca1+cKit+CD150-CD48-). (Two-way ANOVA; * *p* < 0.05). (**E**) Representative FACS plots of PB HSPCs after mobilizing agents’ injection. G-CSF was measured 1 day after the last injection and plerixafor 1 h after injection. Quantification of HSPCs in PB is depicted in the graph. (One-way ANOVA; ** *p* < 0.01, **** *p* < 0.0001). (**F**) Mice were conditioned with busulfan (low and high dose), G-CSF or the combination G-CSF+low-dose busulfan and analyzed 24 h after the last injection (3 mice/group). Upper graph: Total BM cell count after conditioning. (One-way ANOVA; ** *p* < 0.01). Middle graph: Frequency of HSPC (LSK; Lin-Sca1+cKit+) cells in BM 24 h after conditioning, normalized to control mice. (One-way ANOVA; * *p* < 0.05, ** *p* < 0.01). Lower graph: Frequency of long-term HSCs, HPC and MPP cells. (Two-way ANOVA; ** *p* < 0.01). (**G**) Mice were conditioned with busulfan (low and high dose), plerixafor or the combination plerixafor+low-dose busulfan and analyzed after the last busulfan injection or 1 h after plerixafor administration (3 mice/group). Upper graph: Total BM cell count after conditioning. (One-way ANOVA; ** *p* < 0.01). Middle graph: Frequency of HSPC (LSK; Lin-Sca1+cKit+) cells in BM 24 h after conditioning, normalized to control mice. (One-way ANOVA; * *p* < 0.05). Lower graph: Frequency of long-term HSC, HPC and MPP cells. (Two-way ANOVA; ** *p* < 0.01).

The principal purpose of conditioning is to make space in BM before transplantation to improve HSC engraftment and immune recovery. Our aim was to reduce the dose of busulfan used, without compromising immune recovery, by combining a low dose of busulfan with mobilizing agents. G-CSF (granulocyte colony-stimulating factor) and plerixafor are clinically used mobilizing agents to collect HSC cells directly from PB instead of BM. We therefore investigated the effect of busulfan, G-CSF and plerixafor as single agents, and G-CSF or plerixafor in combination with low-dose busulfan on the HSC compartment of NSG mice (three mice/group) 24 h after the last injection of G-CSF and busulfan and 1 h after plerixafor. High-dose busulfan resulted in a significant reduction of total BM cells (Figure 3A). Spleen cell numbers and viability were also significantly compromised with the highest dose of busulfan (Figure 3B). In addition, only the high-dose busulfan showed a reduction of the HSPC population (named LSK in mice; lineage-Sca1+ckit-) in NSG mice (Figure 3C), mostly explained by the decrease of hematopoietic progenitor cells (HPC; Lin-Sca1+cKit+ CD48+) and to a lesser extent multipotent progenitor cells (MPP; Lin-Sca1+cKit+CD150-CD48-) in BM but no long-term HSCs (Lin-Sca1+cKit+CD150+CD48-) (Figure 3D).

The mobilizing efficiency to peripheral blood of G-CSF and plerixafor was tested on NSG mice (three mice/group) as previously published for different mouse strains and with doses adjusted to the NSG mouse strain (Supplementary Figure S2) [22,29,30,31]. An increased HSPC (LSK) population was detected in PB of NSG mice treated with G-CSF (total 250 µg/kg) or plerixafor (10 mg/kg) 24 h or 1 h after the last injection, respectively (Figure 3E). In addition, in accordance with Winkler et al. (2012) [22], the count in PB greatly increased after G-CSF administration due to the increased release of myeloid cells to the periphery (Supplementary Figure S2A). Knowing that G-CSF and plerixafor are able to mobilize HSPCs in NSG mice, we analyzed their effect directly in the BM. G-CSF alone or in combination with the low-dose busulfan had no impact on BM cellularity (Figure 3F, upper graph). However, significant decrease of the HSPC (LSK) compartment was observed after G-CSF treatment, even more prominent than the decrease induced by the high-dose busulfan (Figure 3F, middle graph). As for high-dose busulfan, this decrease was mainly explained by a reduction of the progenitor compartment, but not of long-term HSCs (Figure 3F, lower graph). In contrast, total BM cells were reduced by plerixafor comparable to high busulfan dose (Figure 3G, upper graph). Although no significant decrease of the total HSPC (LSK) population was detected, an interesting but significant reduction of the long-term HSCs as well as MPPs was observed in mice treated with the combination of plerixafor and low-dose busulfan (Figure 3G, lower graph).

In summary, high-dose busulfan and G-CSF administration alone showed consistent reduction in the number of progenitor cells in BM. However, while the low-dose busulfan did not impact the HSPC population in BM, interesting effects were observed when combined with plerixafor, the only condition agent leading to a potential reduction of long-term HSCs.

### 3.4. Long-Term Immune Recovery after Reduced Busulfan Conditioning

**Figure 4:**
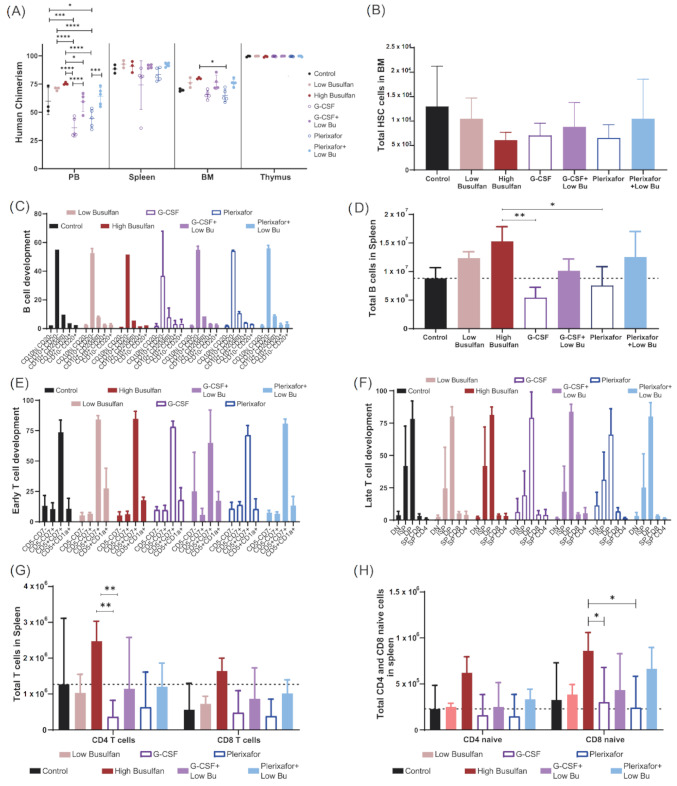
Long-term immune recovery after reduced busulfan conditioning. NSG mice (5 mice/group) were preconditioned with different conditioning regimens (low-dose busulfan, high busulfan, G-CSF, G-CSF+low-dose busulfan, plerixafor and plerixafor+low-dose busulfan) and transplanted with 100,000 CD34 enriched cells from cord blood. (**A**) Achieved human chimerism (% hCD45 cells) in PB, spleen, bone marrow (BM) and thymus 20 weeks after transplantation. (Two-way ANOVA; * *p* < 0.05, *** *p* < 0.001, **** *p* < 0.0001). (**B**) Total number of human hematopoietic stem cells (HCS) in NSG BM 20 weeks after transplantation. (**C**) Proportion of B-cell developmental stages in BM in the different conditioned regimen groups. (**D**) Total B cell counts in spleen 20 weeks after transplantation. (One-way ANOVA; * *p* < 0.05; ** *p* < 0.01). (**E**,**F**) Proportional T-cell developmental stages (early and late) in the thymus in the different conditioning regimen groups. (**G**) Total T-cell numbers (CD4+ and CD8+ cells) in spleen 20 weeks after transplantation. (Two-way ANOVA; ** *p* < 0.01). (**H**) Total naïve T-cell numbers (CD4+ and CD8+ cells) in spleen 20 weeks after transplantation. (Two-way ANOVA; * *p* < 0.05).

Finally, we aimed to study if the direct effects of the different conditioning regimens on the cellular composition of the BM would also lead to better engraftment in vivo after CD34 transplantation. NSG mice (five mice/group) were preconditioned with different conditioning regimens (low-dose busulfan, high-dose busulfan, G-CSF, G-CSF+low-dose busulfan, plerixafor and plerixafor+low-dose busulfan) and transplanted with 1 × 10^5^ CD34/kg enriched cells from cord blood. As previously described, human chimerism increased with increasing busulfan dose. Combining low-dose busulfan with either of the mobilizing agents did not increase human chimerism, achieving similar engraftment to low-dose busulfan only. In addition, G-CSF or plerixafor alone yielded lower human chimerism in BM (Figure 4A and Supplementary Figure S3). However, no significant differences in the number of human HSC engrafted cells in BM were detected across the conditions (Figure 4B). B-cell development in BM was consistent across all conditions (Figure 4C); however, a lower number of B cells was observed in spleen of mice conditioned with the single mobilizing agents. In addition, no difference was observed between the combinations and the low-dose busulfan group (Figure 4D). In parallel, T-cell development in the thymus was uniform across all conditions, both in early (Figure 4E) and late developmental stages (Figure 3F). The T-cell output, both CD4+ and CD8+ T cells, was significantly lower after single G-CSF or plerixafor conditioning and no improvement was observed with the combinations compared to using only low-dose busulfan (Figure 4G). Only an enhanced naive T-cell compartment, most prominent for CD8+ naive cells, was detected by combining plerixafor with a low dose of busulfan (Figure 4H).

Hence, single mobilizing agents did not yield sufficient immune reconstitution in NSG mice by themselves. In addition, the combination of a low dose of busulfan with mobilizing agents did not reveal additive effects, and reconstitution efficiency was primarily driven by busulfan. Only the naïve T-cell compartment seemed to be boosted by plerixafor. None of the novel combinations reached high-dose busulfan reconstitution levels. However, plerixafor apparently could have more impact on the lymphoid progenitors than on the myeloid, which could be interesting to further investigate from a clinical perspective**.**

## 4. Discussion

The NSG mouse model is suitable to study in vivo detection and quantification of HSCs and human immune cells, and can therefore be used to evaluate the effects of stem cell-based therapies. Preconditioning of mice prior to human HSC transplantation is important to ensure successful homing and HSC development. In murine preclinical experiments, the most commonly used conditioning regimen is based on total body irradiation (TBI; x-rays or γ-rays). However, the irradiation procedure induces high stress levels and intestinal damage in the mice, and leads to weight loss and potentially death of the animal on some occasions. Therefore, it is critical to maintain irradiated mice under strict aseptic conditions and continuous health control. In addition, mice can absorb different doses of irradiation depending on their weight and position during the procedure, resulting in a heterogenous group of conditioned mice. Alternative conditioning with chemotherapy drugs such as busulfan, which is commonly used in human HSC transplantation, represents a suitable alternative offering a simple, convenient, individual, weight-adjusted and less-toxic conditioning regimen. Busulfan is indeed an attractive and effective alternative conditioning model that allows an improved human immune reconstitution and better well-being and survival of the mice, which is highly important when working with precious patient material.

Although previous groups already set the most suitable dose of busulfan to condition NSG mice [23,24,27,28], we present here a more extensive analysis of the thymus and T-cell development, leading to higher T cells in the periphery after dosing with busulfan compared to TBI as identified also by Choi et al. [24]. A more preserved and less damaged thymic tissue after busulfan conditioning compared to TBI may explain the higher T-cell outcome observed. While busulfan may have a more targeted effect on BM, TBI is a general therapy causing damage in thymic and lymphoid tissue that will impact T-cell output. A dose of 50 mg/kg busulfan (split in two doses 24 h apart) provides optimal human cell engraftment not only in NSG mice, but also for other immunodeficient mice like Rag2^−/−^ or Rag1^−/−^ [32]. Normal human B- and T-cell development was obtained also with lower doses of busulfan, but the output of B and T cells in the periphery was dose dependent. Chevaleyre et al. (2013) [23] described that although increasing human CD45 chimerism was observed with increasing doses of busulfan (as we also described), no impact on the number of colony-forming cells was detected, which would explain the B- and T- cell developmental pattern we observed across the conditions.

The direct effect of busulfan and mobilizing agents used in this study (G-CSF and plerixafor) on BM and HSPC populations was analyzed in NSG mice. To the best of our knowledge, G-CSF and plerixafor have not been used previously in the NSG mouse model. Therefore, G-CSF and plerixafor doses were derived from published literature on other mouse strains [22,29,31] and the HSC mobilizing capacity was analyzed on PB of the NSG mice. NSG mice showed a significant capacity to mobilize HSC to the periphery after G-CSF or plerixafor administration. While busulfan and G-CSF affected more mature progenitor populations such as HPC and MPP in BM, plerixafor boosted the reduction in BM and increased mobilization of long-term HSCs.

Although interesting effects on different HSPC populations were observed in BM shortly after administration, the longer-term human cell engraftment and immune development after CD34 transplantation did not reflect that direct effect. G-CSF and plerixafor alone allowed appropriate immune development as described previously by Huston et al. [29]. However, when combined with low-dose busulfan, no additive effect was observed between the mobilizing agents and the chemotherapy. The main parameters of chimerism and immune development observed in the combination groups were comparable to those of the low-dose busulfan group, meaning that immune reconstitution was triggered by the chemotherapy conditioning rather than the non-chemotherapy agents. Only the naive T-cell compartment tended to be improved by the addition of plerixafor to low-dose busulfan, which could be caused by the effect of plerixafor on the long-term HSC cells in BM. More extensive pharmacokinetic and pharmacodynamic studies of busulfan, G-CSF and plerixafor in NSG mice will help to select the most suitable doses and timings to ensure a proper model for humanized mice. As G-CSF and plerixafor are clinically approved as mobilizing agents, a small trial with patients has been already performed where patients were preconditioned with a myeloablative regimen together with G-CSF and plerixafor prior to transplantation. No suitable engraftment was achieved with a minimal myeloablative regimen [33]; however, the addition of G-CSF and plerixafor to a TCRαβ+/CD19+-depletion regimen appears to solve the problem of graft failure after HSC transplantation, with no additional risks of toxic complications and associated morbidity [34].

Notably, the conditioning field is moving towards antibody-based conditioning that will target and potentially deplete stem cells without causing off-target toxicity. Antibody-based conditioning regimens are being developed, which may ultimately achieve long-term myeloid engraftment without the associated toxicities of current chemotherapy-based regimens. Different variations of antibody-based conditioning are being tested both preclinically and clinically, such as antibody-drug conjugates specifically targeting HSPCs. Antibody-drug conjugates (ADC) such as CD177-ADC [35,36,37] or CD45-ADC [38,39,40,41,42] have proven to be a safer conditioning regimen than conventional chemotherapy in preclinical models. In addition, monoclonal antibodies targeting CD117 [43,44,45,46] have been successfully developed and paved the way for the use of the anti-CD117 antibody in a currently ongoing clinical trial (NCT02963064).

Less toxic and more directed conditioning regimens are needed to improve outcomes of all allogeneic and autologous gene therapy stem cell transplantations. The possible implications of these improvements are substantial and could potentially impact allogeneic and autologous transplants worldwide.

## Data Availability

The data presented in this study are available on request from the corresponding author. The data are not publicly available due to use of human cord blood samples.

## Supplementary Materials

The following are available online at https://www.mdpi.com/article/10.3390/cells10051077/s1, Figure S1: Human immune reconstitution kinetics after different dose busulfan condition in NSG mice, Figure S2: Mobilizing agents (G-CSF and Plerixafor) dosage on NSG mice; Figure S3: Human immune reconstitution after different conditioning regimens in NSG mice.
