# Supplementary material for: Combining Mobilizing Agents with Busulfan to Reduce Chemotherapy-Based Conditioning for Hematopoietic Stem Cell Transplantation
Source: Cells. 2021 Apr 30;10(5):1077. doi: 10.3390/cells10051077 (PMC8147230; doi:10.3390/cells10051077)
Supplement: Supplementary file 1 [file cells-10-01077-s001.zip › cells-1179767-supplementary.pdf]

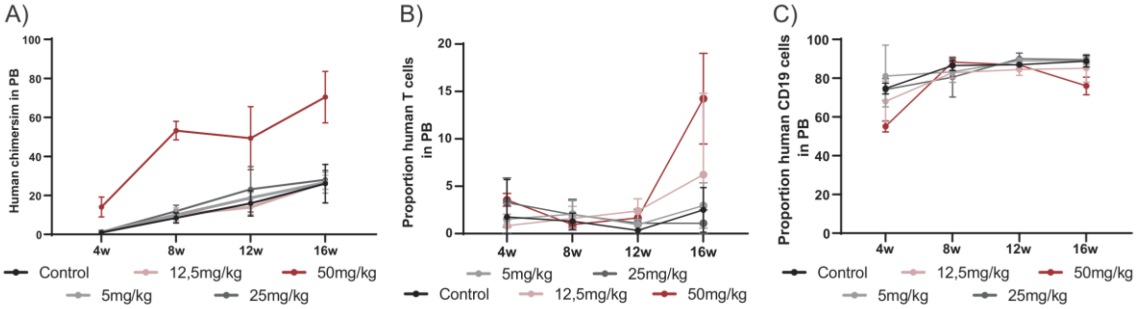

Supplementary Figure 1. Human immune reconstitution kinetics after different busulfan dose conditioning in NSG mice. Human chimerism kinetics, B) human T-cell development kinetics and C) Human B-cell development kinetics over time in PB of CD34 transplanted NSG mice after pre-conditioning with different doses of busulfan (Control, 5mg/kg, 12,5mg/kg, 25mg/kg and 50mg/kg).

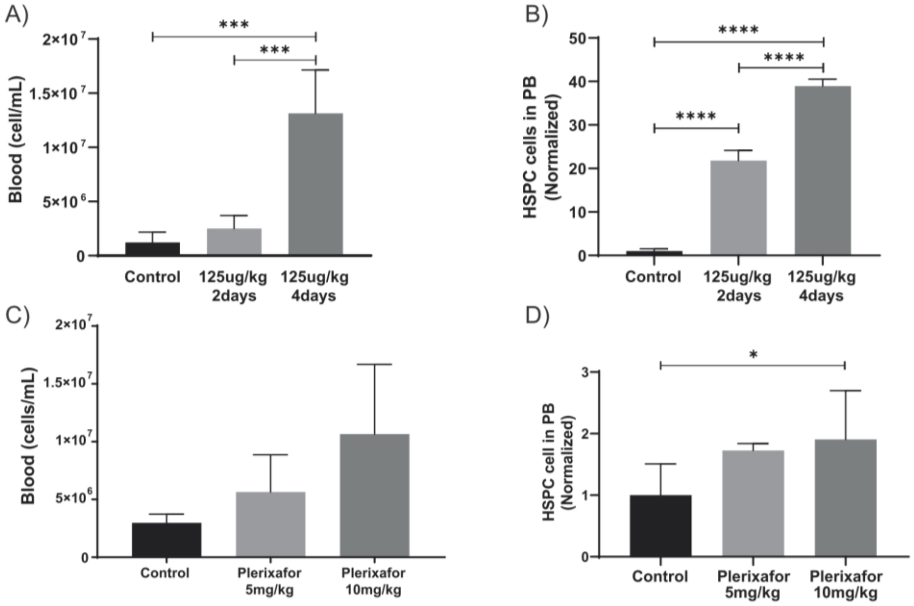

Supplementary Figure 2. Mobilizing agents (G-CSF and Plerixafor) dosage in NSG mice. Effect of different doses of G-CSF in mice A) PB cell numbers and B) HSPC mobilization capacity. Effect of different doses of G-CSF in mice C) PB cell numbers and D) HSPC mobilization capacity. (One-way ANOVA; *p<0,05; **p<0,01; ***p<0,001; ****p<0,0001).

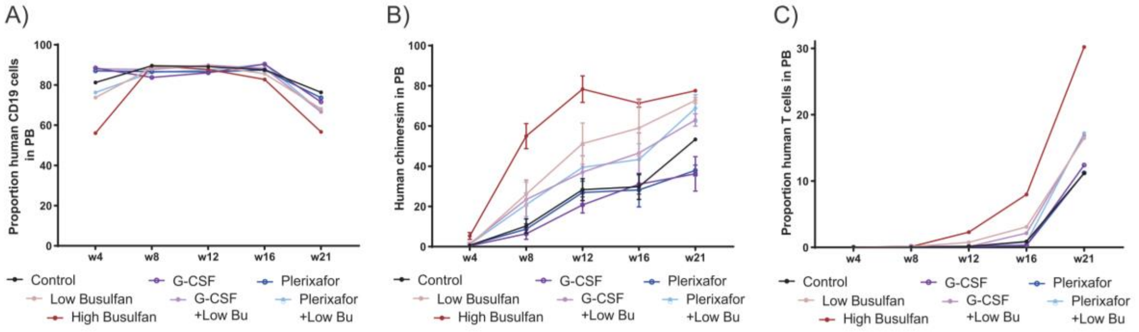

Supplementary Figure 3. Human immune reconstitution kinetics after conditioning regimens in NSG mice. A)Human chimerism kinetics, B) human T-cell development kinetics and C) human B-cell development kinetics over time in PB of CD34 transplanted NSG mice after pre-conditioning with different regimens (Control, low dose busulfan, high busulfan, G-CSF, G-CSF+low dose busulfan, Plerixafor and Plerixafor+low dose busulfan).
